# Impact of Novel Sorghum Bran Diets on DSS-Induced Colitis

**DOI:** 10.3390/nu9040330

**Published:** 2017-03-27

**Authors:** Lauren E. Ritchie, Stella S. Taddeo, Brad R. Weeks, Raymond J. Carroll, Linda Dykes, Lloyd W. Rooney, Nancy D. Turner

**Affiliations:** 1 Intercollegiate Faculty of Genetics, Texas A&M University, College Station, TX 77843, USA; lizabethritchie@gmail.com; 2 Nutrition & Food Science Department, Texas A&M University, College Station, TX 77843, USA; taddeostella2@gmail.com; 3 Department of Veterinary Pathobiology, Texas A&M University, College Station, TX 77843, USA; BWEEKS@cvm.tamu.edu; 4 Department of Statistics, Texas A&M University, College Station, TX 77843, USA; raymond9620@gmail.com; 5 Department of Soil and Crop Sciences, Texas A&M University, College Station, TX 77843, USA; linda.dykes@ars.usda.gov; 6 Soil and Crop Sciences Department, Texas A&M University, College Station, TX 77843, USA; lrooney@tamu.edu

**Keywords:** colon, gene expression, inflammatory bowel disease, dietary fiber, polyphenols, short chain fatty acids, *Sorghum bicolor*

## Abstract

We have demonstrated that polyphenol-rich sorghum bran diets alter fecal microbiota; however, little is known regarding their effect on colon inflammation. Our aim was to characterize the effect of sorghum bran diets on intestinal homeostasis during dextran sodium sulfate (DSS)-induced colitis. Male Sprague-Dawley rats (*N* = 20/diet) were provided diets containing 6% fiber from cellulose, or Black (3-deoxyanthocyanins), Sumac (condensed tannins) or Hi Tannin Black (both) sorghum bran. Colitis was induced (*N* = 10/diet) with three separate 48-h exposures to 3% DSS, and feces were collected. On Day 82, animals were euthanized and the colon resected. Only discrete mucosal lesions, with no diarrhea or bloody stools, were observed in DSS rats. Only bran diets upregulated proliferation and *Tff3*, *Tgfβ* and short chain fatty acids (SCFA) transporter expression after a DSS challenge. DSS did not significantly affect fecal SCFA concentrations. Bran diets alone upregulated repair mechanisms and SCFA transporter expression, which suggests these polyphenol-rich sorghum brans may suppress some consequences of colitis.

## 1. Introduction

Inflammatory bowel disease (IBD), which includes Crohn’s disease and ulcerative colitis (UC), affects nearly 1.4 million people in the United States [1]. UC is characterized by varying degrees of mucosal inflammation that extends proximally from the rectum, and the sites of inflammation are commonly associated with mucin depletion and loss of epithelial barrier integrity [2]. Although the complete etiology of UC is not fully understood, recent research has indicated that perturbations in host immune response to intestinal microbiota could be a major factor in the progression of this disease [2,3].

The intestinal microbiota plays a role in host health by affecting the luminal environment and interacting with host epithelial cells. Microbial impacts on health can be derived from fermentation of dietary fiber, alteration of intestinal motility and pH, as well as protection against invading pathogens and improving the integrity of tight junctions [4,5]. Fermentation of dietary fiber produces metabolites including short chain fatty acids (SCFA), such as butyrate, which is the preferred fuel source for colonocytes [6,7]. SCFA also affects gene expression, cellular differentiation and has anti-inflammatory activity in the colon epithelium [6,8,9,10,11]. The microbiota affects host innate immunity by signaling through toll-like receptors (TLR) and the nuclear factor-κB (NF-κB) pathway [4,12]. Furthermore, dysregulation of the TLR pathway has been associated with the progression of intestinal inflammation and the pathogenesis of UC [4,12].

Repeated bouts of intestinal inflammation can compromise epithelial barrier function, thereby allowing bacteria and antigens to enter the body from the lumen. Therefore, preservation of colonocyte proliferation and expression of proteins involved in epithelial cell migration and barrier restitution, such as transforming growth factor beta (TGFβ) and trefoil factor (TFF3), are crucial for repairing lesions and maintaining epithelial barrier integrity [13,14]. In addition, perturbation of TLR signaling pathways can also affect epithelial cell proliferation and apoptosis [4]. These observations suggest that interventions that normalize these functions could mitigate the damage induced by colitis.

Composition of colonic microbial populations can be altered by numerous environmental factors, including dietary constituents [15,16], such as dietary fiber type and plant polyphenols [17,18,19,20,21,22]. Recent in vivo studies have reported the ability of hydrocaffeic acid, tea polyphenols and tannic acid to differentially affect the microbiota by enhancing certain taxa and reducing others [17,23]. In addition, some sources of dietary polyphenols have been reported to mitigate some of the deleterious effects of colitis, including changes in the intestinal microbiota that occur with colitis. Recent studies have characterized the high concentrations of phenolic molecules present in some sorghum grain varieties [24,25,26]. To our knowledge, this is the first study to analyze the ability of these sorghum brans to suppress an experimental model of UC.

Based on these prior observations, we hypothesized that diets containing insoluble fiber, especially in combination with polyphenols, would mitigate the perturbation of bacterial metabolites and TLR signaling that normally occurs in colonocytes during colitis. Therefore, in order to elucidate the effects of bran derived from sorghum varieties containing various types of polyphenols (3-deoxyanthocyanins, condensed tannins or both polyphenols) on the deleterious effects of UC, we determined fecal SCFA concentrations, TLR signaling, colonocyte gene expression and epithelial cell proliferation, apoptosis and epithelial barrier maintenance during repeated DSS exposures.

## 2. Materials and Methods

### 2.1. Animals and Diets

The animal use protocol was approved by the University Animal Care Committee of Texas A&M University. Eighty male Sprague-Dawley rats (Harlan Sprague-Dawley, Houston, TX, USA) were stratified by body weight and assigned to receive one of four experimental diets (*N* = 20/diet) at 40 days of age. Diets were prepared to contain similar levels of macronutrients and similar types of dietary fiber so that the major differences between the diets primarily would be the bioactive compounds. The four diets contained 6% dietary fiber from (1) cellulose, or bran isolated from sorghum grains that contain: (2) high levels of 3-deoxyanthocyanins (Black bran), (3) high levels of condensed tannins and low levels of 3-deoxyanthocyanins (Sumac bran) or (4) both 3-deoxyanthocyanins and condensed tannins (Hi Tannin Black bran). Antioxidant capacity, total polyphenol content, tannin content [25] and proportions of soluble and insoluble fiber (Experiment Station Chemical Laboratories, University of Missouri) were quantified for each experimental diet (Table 1) [27].

After 21 days of experimental diets, half of the rats were exposed to three sequential 48 h dextran sodium sulfate (DSS, 36,000–50,000 MW) (MP Biomedicals, Irvine, CA, USA) treatments (3% DSS) in distilled drinking water, with 12 d between each DSS exposure. Between DSS exposures, water was provided to mimic remissions in human chronic UC patients [28]. The remaining half of the animals received distilled drinking water throughout the course of the study.

Body weight, food intake and overall animal health were routinely monitored. Rats were weighed upon arrival (Day 1), prior to beginning experimental diets (Day 19), 48 h before each DSS exposure and prior to termination (Day 82). Food intake was measured prior to the first DSS treatment (Day 33), following the second DSS treatment (Day 56) and prior to termination (Day 77, following third DSS treatment). On Day 82, rats were euthanized by CO_2_ asphyxiation.

### 2.2. Fecal Collections and Processing

Fresh fecal samples were collected prior to DSS exposure, 48 h after each DSS exposure and prior to termination. Moisture content was used as a surrogate measure for disease severity and was determined by drying samples at 60 °C until weights became static.

#### Short Chain Fatty Acid Analysis

Feces were collected immediately following defecation, transferred to cryotubes and snap-frozen in liquid nitrogen. Frozen samples were ground and SCFA extracted and quantified by comparison to mixed standards using a Varian 3900 gas chromatograph (Walnut Creek, CA, USA) as described previously [29]. SCFA concentrations were multiplied by the 24 h fecal weights to calculate daily SCFA excretion.

### 2.3. Tissue Fixation for Immunohistological Processing

After fecal material was removed from the colon, two 1-cm segments from the distal end were rinsed with RNase free phosphate-buffered saline (PBS) and fixed in either a 4% paraformaldehyde or 70% ethanol solution (for TUNEL and PCNA assays, respectively) prior to embedding in paraffin.

### 2.4. Mucosal Samples

The remaining colon was washed twice in RNase-free PBS and the mucosa removed by scraping on a chilled RNase free surface. An aliquot of the scraped mucosa was transferred to an RNase-free homogenization tube along with 500 μL of denaturation solution (Ambion, Austin, TX, USA) and homogenized. Following homogenization, the aliquot was stored at −80 °C [30]. The remaining aliquot was homogenized in 400 μL of protein buffer, centrifuged (15,000× *g* 30 min at 40 °C), and the supernatant was stored at −80 °C [31].

### 2.5. Inflammation and Injury Histological Scores

The degree of inflammation and morphological injury caused by DSS exposure was assessed in a blinded manner using H&E-stained tissues. The extent of inflammation (score of 0–3) and epithelial injury (score of 0–3) were graded as described previously [32,33].

### 2.6. Activated NF-κB Measurement

Activated NF-κB was assessed using the manufacturer’s protocol for whole cell lysates of mucosa with the TransAM™ NF-κB Chemi Lysis assay (Active Motif, Carlsbad, CA, USA). Activated NF-κB (p50/p65 subunits) extracted from each sample was added to an oligonucleotide-coated plate, bound to a primary antibody and identified with a secondary antibody. A nuclear extract of Jurkat cells was used as a positive control for the p65 NF-κB subunit, and protein buffer and lysis buffer were run as negative controls.

### 2.7. Immunohistological Measurement of Proliferation and Apoptosis

Colonocyte proliferation was measured using a monoclonal antibody to proliferating cell nuclear antigen (PCNA; Anti-PC-10, Covance, Emeryville, CA, USA). The total number of proliferating cells, the position of the highest proliferating cell and the total cell number/crypt column (crypt column height) were assessed in 25 crypt columns/rat, as described previously [30,31,34].

Paraffin sections of the 4% PFA fixed tissues were used to measure apoptosis with the TUNEL assay. Total numbers of apoptotic cells were determined in 50 crypt columns/rat, and an apoptotic index (apoptotic cells/crypt column height) was determined as described previously [31,34].

### 2.8. Measurement of Gene Expression Using Real-Time PCR

Total RNA was isolated from mucosal samples using Phase Lock Gel™ tubes (5 Prime, Gaithersburg, MD, USA) and the ToTALLY RNA™ Kit (Ambion, Austin, TX, USA) followed by DNase treatment (DNA-*free*™ Kit, Ambion, Austin, TX, USA). RNA quality was assessed using an Agilent Bioanalyzer, and mRNA concentrations were measured using spectrophotometry. First strand cDNA was synthesized using random hexamers, oligo dT primer (Promega, Madison, WI, USA) and Superscript™ III Reverse Transcriptase following the manufacturer’s instructions (Invitrogen, Carlsbad, CA, USA).

Real-time PCR was performed using an ABI 7900 HT thermocycler (Applied Biosystems, Foster City, CA, USA) on select genes (*Tollip*, *Tff3*, *Zo1*, *Slc16a1*, *Il6*, *Il1β*, *Il12b*, *Tnf*α, *Slc5a8*, *Tgfβ*, *Tlr4*, *Tlr2*, *Myd88*, *Rela/p65*, *Cox2*) (Table S1). Selected primers were pre-loaded into Taqman^®^ Array Micro Fluidic Cards (Applied Biosystems, Foster City, CA, USA) and used for subsequent qPCR analysis. Expression levels were normalized to 18S gene expression.

### 2.9. Statistical Analysis

Data were analyzed using two-way analysis of variance (ANOVA) including variables of diet, DSS treatment, and their interaction in SAS 9.1 (SAS Institute, Inc., Cary, NC, USA), in which a *p*-value of < 0.05 was considered significant. Sample size in each experimental group for all analyses is *N* = 10, except for relative expression of selected gene targets (i.e., *Tollip*, *Tff3*, *Slc16a1*, *Il6*, *Il12b*, *Slc5a8*, *Rela/p65*) and 24 h SCFA production on Day 72. Relative expression of the aforementioned targets was greater than 2.5 SD from the mean for one animal in the Sumac DSS group, and therefore, that observation was excluded from further analysis. Sample size for 24 h SCFA production on Day 72 was *N* = 9 for all experimental groups since 24 h dry weight measurements for one animal set were not taken at this time point. All data are reported as least squares (LS) means ± standard error of the mean (SEM). Linear regression was calculated for the change in colonic injury and mucosal NF-κB activity between control rats (not treated with DSS) and DSS-treated rats.

## 3. Results

### 3.1. Body Weight, Intake and Fecal Moisture Content

We hypothesized that feeding sorghum bran diets would attenuate the effects of DSS-induced UC in rats. Body weight, food intake and fecal moisture were assessed to monitor disease severity and ensure that the experimental diets were adequately consumed. There were few differences in body weight or food intake noted among the groups. The Hi Tannin Black bran control rats weighed more on Day 37 (*p* = 0.03), Day 39 (*p* = 0.03), Day 53 (*p* = 0.03) and Day 58 (*p* = 0.04) than the cellulose control rats (Table S2). On Day 33 (prior to DSS#1), we observed a slightly lower food intake in cellulose DSS rats compared to Sumac DSS rats (*p* < 0.05); however, there were no significant differences in intake for any experimental groups on Day 56 (post DSS#2) or Day 77 (post DSS #3) (Table S3).

Fecal moisture content in bran-fed rats (i.e., Black, Sumac and Hi Tannin Black) was significantly higher than cellulose-fed rats in all samples. Fecal moisture contents did not markedly increase following DSS treatment for any diet group (<6%), except for samples from rats consuming the Sumac diets on Day 44 (post DSS #1) and Day 58 (post DSS#2) (Table 2).

### 3.2. Immunohistochemical Analysis of Distal Colon

**Figure 1:**
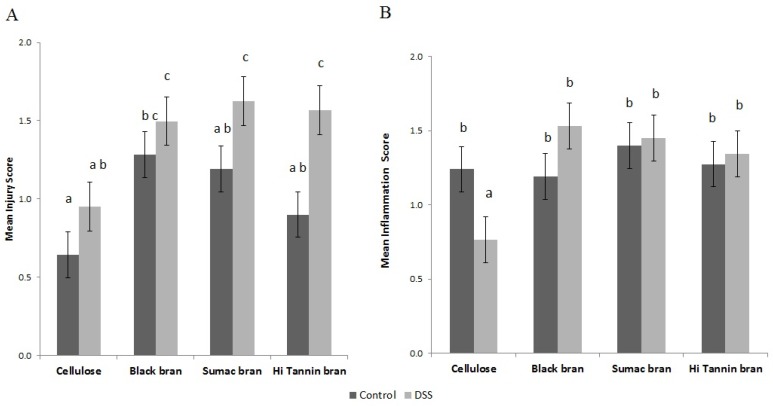
Colon injury scores (**A**) and inflammation scores (**B**) in rats consuming diets containing cellulose or Black, Sumac or Hi Tannin Black sorghum bran. Rats were provided water (control) or DSS (3% for 48 h, three times, two-week separation). Values are least squares mean ± standard error of the mean. Means without common superscripts differ (*p* < 0.05). DSS = dextran sodium sulfate.

To assess the severity of DSS-induced UC, distal colon segments were analyzed by a pathologist for epithelial injury and inflammatory cell infiltration. Cellulose control rats had lower injury scores than Black bran controls (*p* < 0.05, Figure 1A). DSS treatment elevated injury scores in all groups, with significantly higher scores observed in Hi Tannin Black and Sumac bran rats compared to their diet matched controls (*p* = 0.0072). DSS-treated rats fed bran diets had significantly higher injury scores compared to cellulose DSS rats (*p* < 0.05). There was no significant difference in inflammation score among the treatment groups, except for the cellulose DSS rats, where inflammation was reduced (Figure 1B) (*p* < 0.05).

DSS-treated rats for all diets had shorter crypt heights compared to the diet-matched control rats (*p* < 0.05, Table 3). The combination of insoluble fiber with polyphenols tended to elevate the proliferative index and zone in DSS rats, whereas DSS tended to lower the proliferative index and zone for rats receiving the cellulose diet. There were no differences in apoptotic index between control and DSS rats for all experimental diets. However, Sumac control rats had a significantly higher index than Hi Tannin Black rats that were treated with DSS, which caused a 60% reduction relative to the Hi Tannin Black rats in the control group (*p* = 0.0208, Table 3).

### 3.3. Fecal SCFA Analysis

Gut microbiota is commonly altered during bouts of inflammation associated with UC. Fecal SCFA concentrations can be used as a functional biomarker of changes to overall microbial metabolism and suggest possible changes to the microbiota [35]. To assess if sorghum-based diets affected fecal SCFA concentrations during experimental UC, we collected feces at various time points throughout the study. Prior to DSS treatments (Day 38), we observed a significant diet effect on 24 h SCFA excretion (µmol/24 h) for all SCFA analyzed (Table 4). The proportion of butyrate was lowest in Sumac fed rats (10%–11%) compared to cellulose, Black and Hi Tannin Black bran diets (18%–21%, 23%–24%, 15%–17%, respectively). We observed similar diet effects for all other collections (Days 44, 66, 72, 81) for propionic, isobutyric, butyric, isovaleric, and valeric acids (Table S4 and Table 4, respectively). Following recovery from DSS#1 and DSS#2, we did not observe a significant effect of DSS treatment on 24 h SCFA excretion, except a significant interaction between diet and DSS exposure on Day 44 (post DSS#1) for isobutyric acid and on Day 66 (post DSS#2) for acetic acid (Table S4).

**Figure 2:**
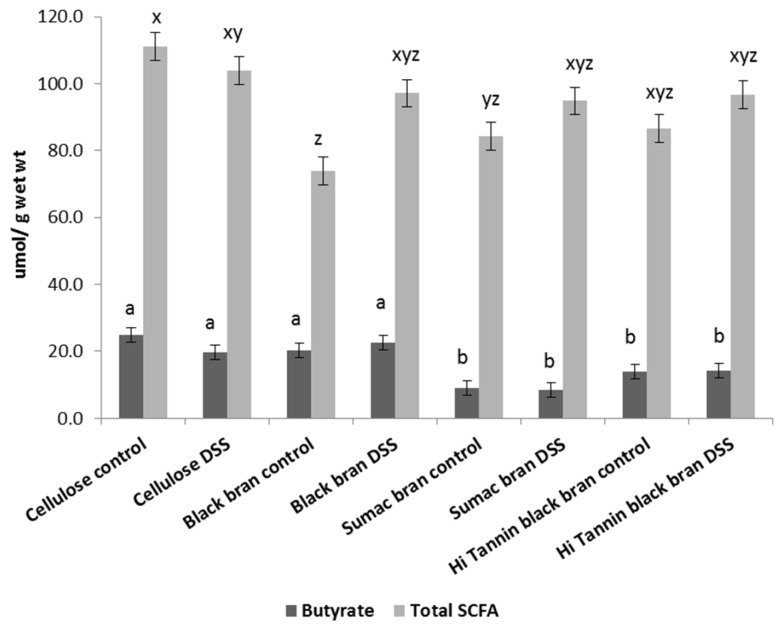
Fecal butyrate and total SCFA concentrations (µmol/g wet weight) collected prior to termination (Day 81). Values are LS means ± SEM. Means without common superscripts differ (*p* < 0.05). DSS = dextran sodium sulfate.

Fecal samples following recovery from all DSS treatments (Day 81) represent the luminal environment closest to termination. On Day 81, we observed the same significant diet effects on 24 h SCFA excretion as observed on Day 38 (prior to DSS treatments) for all SCFA except for acetic and propionic acid (µmol/24 h; *p* < 0.05) (Table 4). Diet significantly affected 24 h butyrate excretion following all DSS treatments (Day 81) with cellulose and Black bran rats having numerically higher amounts (16.4 and 20.1 µmol/24 h, respectively), compared to Sumac and Hi Tannin Black bran rats (8.8 and 14.1 µmol/24 h, respectively). In DSS rats (Day 81), the proportion of excreted butyrate was lower in Sumac bran rats (9%) compared to cellulose, Black or Hi Tannin Black bran rats (19, 23, 15%, respectively) on a wet weight basis (µmol/g), which is similar to the values observed prior to DSS exposure (Figure 2).

### 3.4. Mucosal Gene Expression and NF-κB Activity

**Figure 3:**
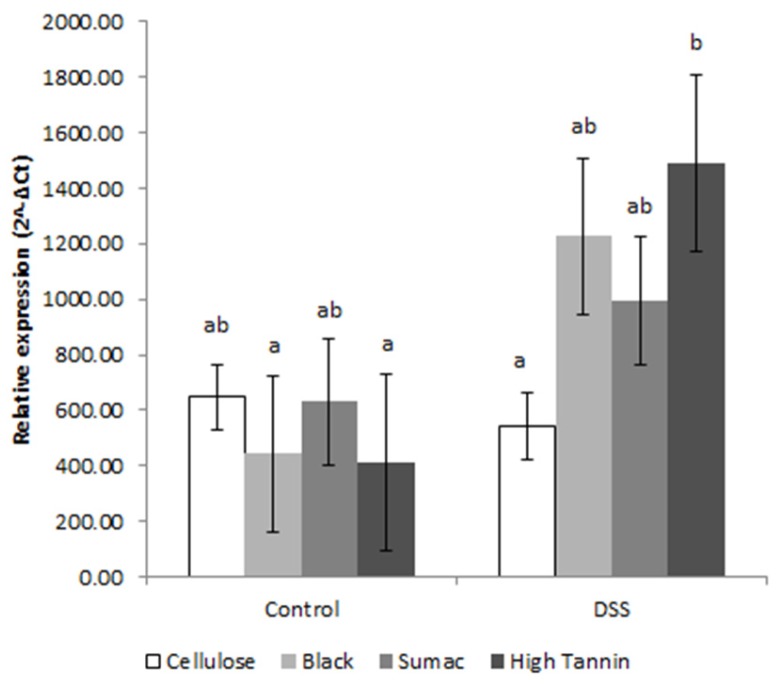
Relative expression (2^−Δ*C*t^) of *Tff3* in scraped mucosa from rats receiving water (control) or DSS treatments (3% for 48 h, three times, two-week separation) to induce colitis. Values are LS means ± SEM. Means without common superscripts differ (*p* < 0.05). See Supplemental Table 5 for actual values. DSS = dextran sodium sulfate. SCFA = short chain fatty acids. Expression levels were normalized to 18S gene expression.

**Figure 4:**
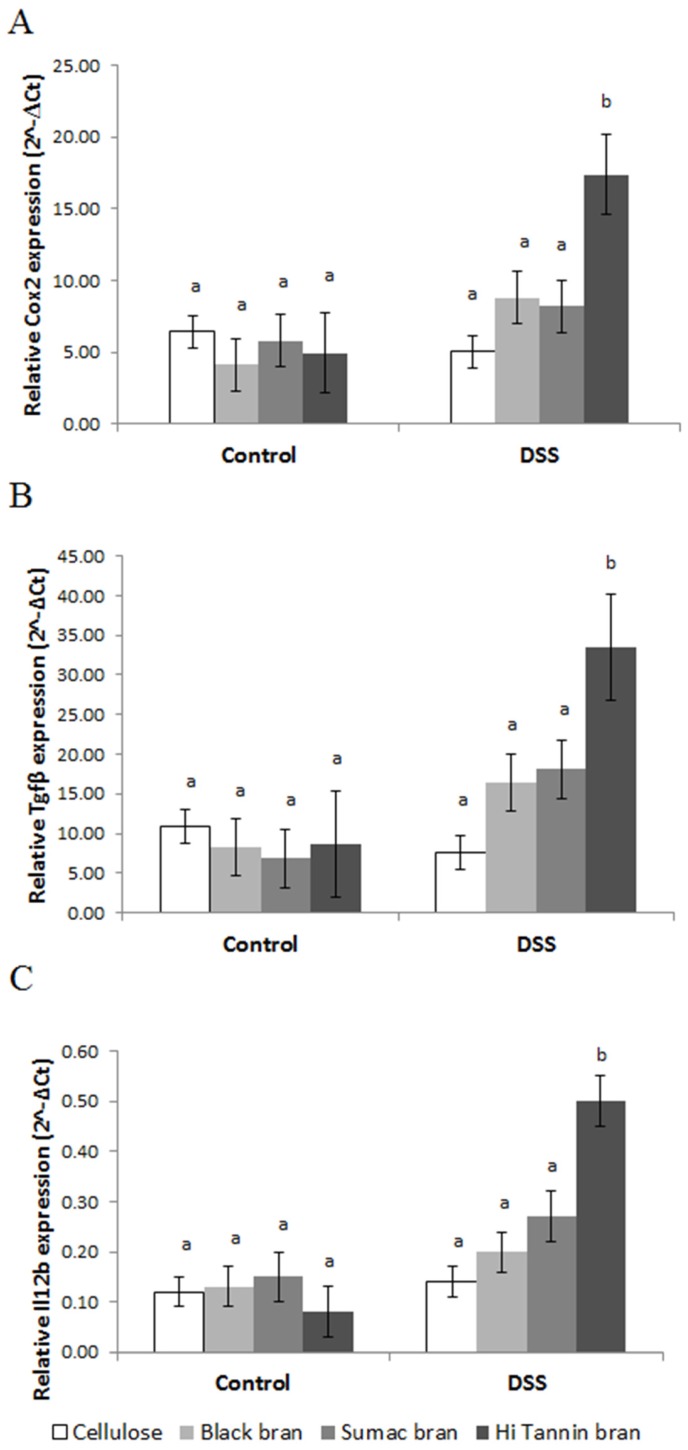
Relative expression (2^−Δ*C*t^) of (**A**) *Cox2*, (**B**) *Tgfβ* and (**C**) *Il12b* in scraped mucosa from rats receiving water (control) or DSS treatments (3% for 48 h, three times, two-week separation) to induce colitis. Values are LS means ± SEM. Means without common superscripts differ (*p* < 0.05). See Supplemental Table 5 for actual values. DSS = dextran sodium sulfate. Expression levels were normalized to 18S gene expression.

UC can affect the expression of TLR signaling in colonic epithelium. To better understand how sorghum brans affected these pathways during DSS-induced UC, we assessed gene expression and NF-κB activity in scraped colonic mucosa. We did not observe any diet or diet*treatment interaction effects for any gene targets except *Cox2* and *Il12b* (diet*treatment effect, *p* = 0.026 and *p* = 0.017, respectively), which are both upregulated by signals derived from NF-κB pathway activation. We observed a significant DSS treatment effect for gene targets involved in the TLR pathway, including *Tlr2*, *Tlr4*, *Myd88*, *Rela/p65* (regulatory subunit of NF-κB), *Tollip*, the cytokines *Tnfα*, *Il12b*, *Il6*, *Cox2*, as well as proteins involved in epithelial barrier restitution *Tff3* and *Tgfβ* (Figure 3 and Table S5, respectively). The relative expression of these targets (except *Tnfα* and *Il12b*) was higher in bran-fed DSS rats and downregulated in cellulose-fed DSS rats compared to their diet matched controls. Relative expression of *Tlr2*, *Tlr4*, *Rela/p65*, *Tollip*, *Cox2*, *Il12b*, *Tff3* and *Tgfβ* was higher in Hi Tannin Black DSS rats, with *Cox2*, *Il12b* and *Tgfβ* having significantly higher expression compared to all other groups (*p* < 0.05; Figure 4).

**Figure 5:**
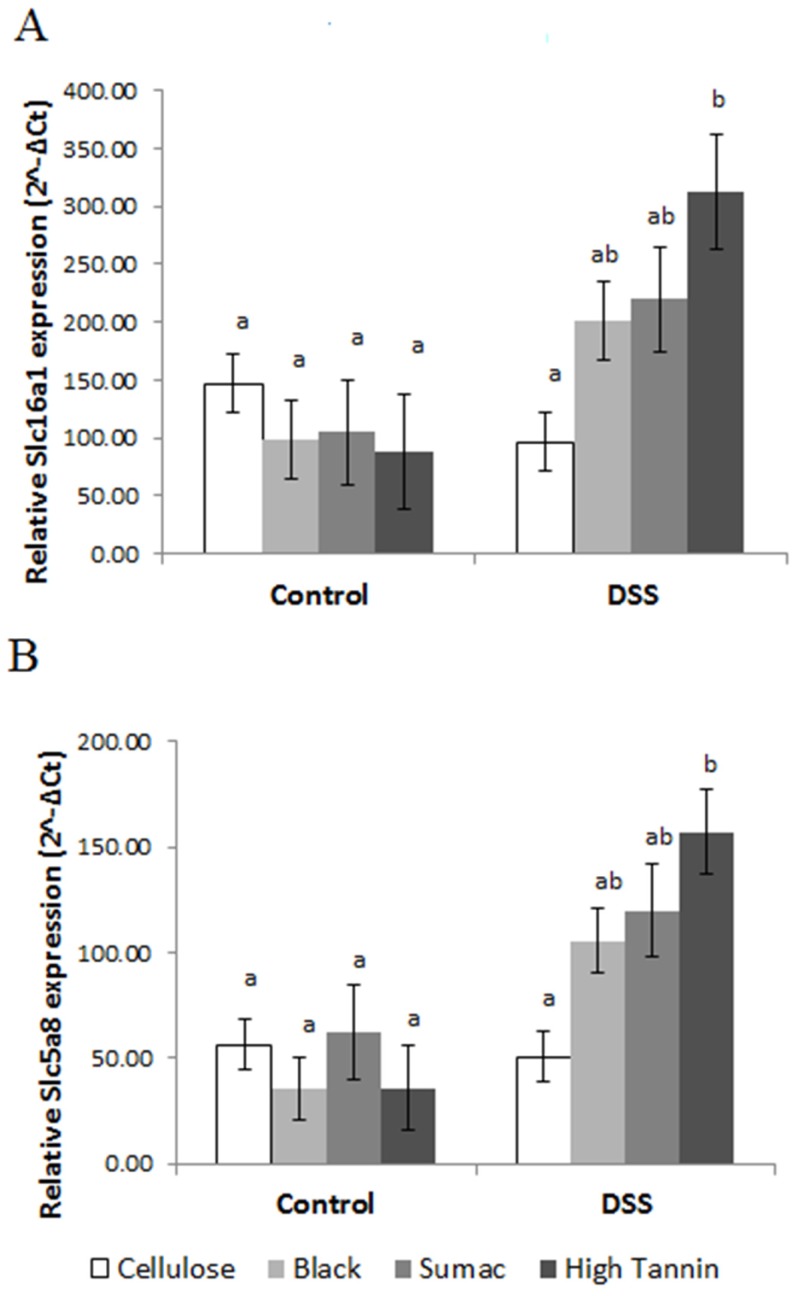
Relative expression (2^−Δ*C*t^) of SCFA transporters (**A**) *Slc16a1* and (**B**) *Slc5a8* in scraped mucosa from rats receiving water (control) or DSS treatments (3% for 48 h, three times, two-week separation) to induce colitis. Values are LS means ± SEM. Means without common superscripts differ (*p* < 0.05). See Supplemental Table 5 for actual values. DSS = dextran sodium sulfate. SCFA = short chain fatty acids. Expression levels were normalized to 18S gene expression.

Relative expression of SCFA transporters *Slc16a1* and *Slc5a8* was significantly higher in Hi Tannin Black DSS rats (*p* = 0.003 and *p* = 0.02, respectively) compared to controls (Figure 5). These expression levels paralleled the differences in total SCFA concentrations observed on Day 81 (prior to termination).

**Figure 6:**
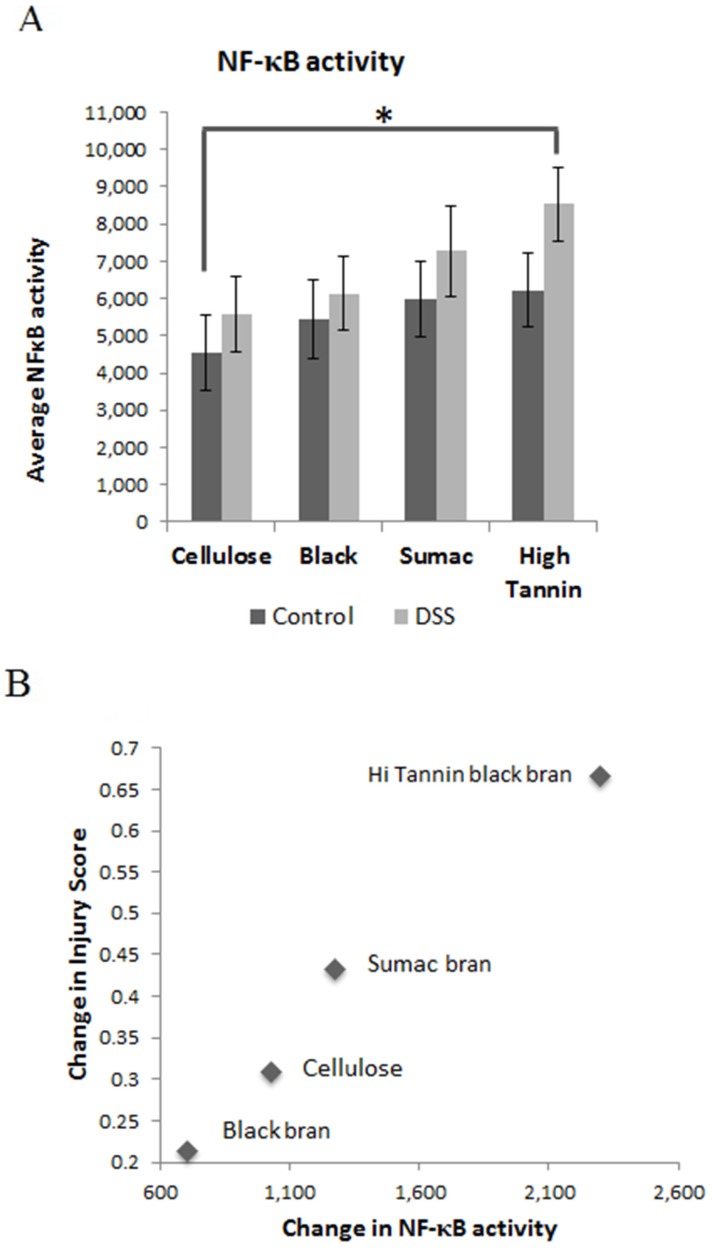
(**A**) NFκB activity (nanograms/20 µL sample) in colonic mucosa from rats receiving water (control) or DSS treatments (3% for 48 h, three times, two-week separation) to induce colitis. Values are LS means ± SEM. (**B**) A linear relationship was observed between the change in injury score and change in NFκB activity (*p* = 0.01, R^2^ = 0.98). DSS = dextran sodium sulfate.

Mucosal NF-κB activity was numerically elevated in DSS-treated rats for all experimental diets, with activity becoming significantly higher in Hi Tannin Black DSS rats compared to cellulose controls (*p* = 0.03; Figure 6A). The relationship between the change in injury score and NF-κB activity induced by DSS was determined to assess whether these changes were unique to the specific diets. We observed a linear relationship between the change in injury score and change in NF-κB activity (*p* = 0.01, *R*^2^ = 0.98), with the Black bran diet resulting in the lowest value compared to cellulose, Sumac and Hi Tannin Black, respectively (Figure 6B).

## 4. Discussion

With the incidence of gastrointestinal diseases, such as UC, on the rise worldwide [36], it is imperative to identify mechanisms to mitigate the onset or progression of chronic intestinal inflammation. Diets containing polyphenols may provide protection against UC due to their ability to alter the intestinal microbiota, as well as provide anti-inflammatory effects and eliminate free radicals [17,19,23]. Additionally, diets rich in fiber have been shown to reduce the risk of developing gastrointestinal disorders [29,37]. Bran isolated from some sorghum varieties provides a primarily non-fermentable fiber source that has high levels of 3-deoxyanthocyanins, condensed tannins or a combination of both compounds [24,26]. The ability of sorghum bran diets to mitigate UC has not been investigated.

We investigated the effect of sorghum bran diets during repeated DSS exposures, a model of recurrent UC [38]. Our observations suggest that disease severity in DSS rats consuming diets containing insoluble fiber was minimal, as we observed no significant differences in body weight, diet intake or marked increases in fecal moisture content following DSS treatments. Maintenance of fecal moisture content during inflammatory bouts could be due to the insoluble fiber content (~95%) of the sorghum bran, which is known to increase fecal bulk, reduce diarrhea and bind carcinogens [39]. Similar to our results, others report insoluble fiber attenuates the deleterious effects of DSS-induced colitis (2%–3.5% DSS for 5–6 d) [40,41]. Although some soluble fibers are known to ease constipation and have been shown to improve symptoms of UC [42], preliminary data from our lab using the same DSS with diets containing 6% pectin, a predominately soluble fiber source, demonstrated significantly higher colonic injury, fecal moisture content and bloody diarrhea [43]. These results suggest that dietary fiber chemical and physical characteristics may directly affect the ability of diet to mitigate the extent of colonic injury and bloody, loose stool associated with DSS colitis (i.e., fibers that are more insoluble and less fermentable in nature are more protective).

Immunohistochemical examination of the distal colon revealed that crypt height was reduced and colonic injury scores elevated in all DSS-treated rats. However, mucosal injury was generally characterized as discrete lesions with only occasional mucosal erosion. One explanation for the minimally elevated injury scores in bran-fed animals compared to cellulose could be due to the larger particle size and structure of the bran, particularly Black bran [44], as compared to the highly refined and small particle size of the purified cellulose. Our observations in DSS animals contrast other studies that utilized similar DSS treatments (3%–5%), which reported mucosal erosion, complete loss of crypts, as well as lesions that extended into the intestinal wall [45]. These data also support the hypothesis that less soluble fibers with reduced fermentability are protective against the damage induced during bouts of colitis, especially as compared to fibers that are readily fermented, such as pectin.

Chronic intestinal inflammation can lead to epithelial barrier dysfunction, and preservation of colonocyte proliferation is crucial for maintaining barrier integrity. Both proliferative index and the proliferative zone were increased at termination (two weeks post DSS#3) for all bran-fed DSS rats compared to controls. The significantly higher proliferative index in Black bran DSS rats may be due to the 3-deoxyanthocyanins in this bran, as other studies have reported that diets containing anthocyanin-rich blueberries reduce epithelial injury, bacterial translocation and inflammation during DSS-induced colitis [46,47]. In addition to maintaining proliferation, proteins responsible for epithelial migration such as *Tgfβ* and *Tff3* allow for restitution of the epithelial barrier following colonic injury [13,14,48]. *Tgfβ* is thought to play a major role in cellular differentiation, migration and wound healing in vitro [48], and Trefoil factor proteins have been shown to be involved in cellular migration and suppression of apoptosis [14]. We observe higher expression of *Tff3* and *Tgfβ* genes in colonic mucosa of DSS rats consuming bran diets compared to their diet matched, non-DSS controls. It has been reported that *Tff3* expression is suppressed in 2,4,6-trinitrobenzenesulfonic acid models of experimental colitis [13], and the absence of *Tff3* expression in DSS induced colitis significantly delays healing of mucosal injury up to 12 d post DSS exposure [49]. Our results demonstrate that sorghum brans were able to eliminate this negative outcome, as we observe elevated expression of *Tff3* even after repeated DSS treatments.

In general, we determined that repeated DSS exposures in sorghum bran-fed rats does not cause drastic mucosal damage as described in similar experimental colitis models [45]. Although injury scores were higher in bran-fed DSS rats, our results indicate that bran diets could have the ability to support the repair of the mucosal damage associated with UC by modulating epithelial cell proliferation and the expression of *Tgfβ* and *Tff3*, which we did not observe in cellulose DSS rats. These results could indicate that the polyphenol content of these sorghum bran diets could be a factor in upregulating epithelial repair mechanisms, which parallels recent studies demonstrating that plant polyphenols can mitigate colonic injury and improve UC symptoms [17,23,50]. Although a direct mechanism has yet to be elucidated, beneficial properties could include modulating the luminal environment and microbiota, which is well documented to directly affect the progression and severity of UC. Therefore, we sought to elucidate how sorghum bran diets can alter the luminal environment and microbiota by analyzing fecal SCFA and mucosal gene expression of the toll-like receptor (TLR) pathway.

The intestinal microbiota has a profound impact on the host immune system, and dysbiosis of these bacterial populations and suppression of SCFA production has been implicated in UC [51,52]. Of particular importance is butyrate, which is not only the preferred metabolic substrate for colonocytes, but has other pleiotropic effects, including anti-inflammatory activity and affecting gene expression and differentiation in epithelial cells [7,53]. We do not observe a significant decrease in fecal butyrate excretion following DSS exposure in rats fed the bran diets. Additionally, we do not observe a significant reduction in the excretion of fecal butyrate or other SCFA (µmol/24 h) in bran-fed rats during an active disease state, which suggests that DSS-induced colitis did not affect microbial fermentation patterns in these animals. In contrast, cellulose DSS rats had significantly lower 24 h butyrate excretion following DSS#2 and DSS#3 compared to their diet matched controls. Although we did not directly measure butyrate absorption, we observe significantly higher relative expression of SCFA transporters, *Slc16a1* and *Slc5a8*, in Hi Tannin Black DSS rats compared to their diet matched controls, which could explain the reduced fecal butyrate observed in these animals. This contrasts previous reports of decreases in SCFA transporter expression in diseased tissues and patients with IBD [54], which is similar to the suppressed SCFA excretion and transporter expression observed in the cellulose DSS rats. Thus, our results may suggest that the properties of the sorghum bran diets could protect against significant suppression of SCFA production and reduced SCFA transporter expression commonly associated with intestinal inflammation and UC [55,56].

Our SCFA observations indicate that there may be differences in the bacterial populations or their metabolism between experimental groups, which we confirmed in a separate study characterizing the microbiota of feces collected post DSS #2 and DSS #3. Results indicated that diet significantly affected all bacterial genera following both DSS treatments and that diets containing tannins (i.e., Sumac and Hi Tannin Black) significantly affected species richness compared to cellulose DSS rats [20]. Other studies have also determined that anthocyanins and hydrolysable tannins from other dietary constituents have differential effects on intestinal bacterial populations [50] and that dietary fiber has an effect on intestinal microbiota richness [21,22].

The microbiota also plays a major role in the status of the epithelial barrier by interacting with TLR, which provides homeostatic interactions between the microbiota and host. However, in a disease state, this pathway has been shown to upregulate the immune response, as well as cause hyper-proliferation, which increases the risk of tumorigenesis [57]. Studies have shown that experimental models of UC and patients with chronic intestinal inflammation have differential TLR expression [58]. In our study, we observe a significant effect of DSS treatment on *Tlr2* and *Tlr4* and other targets associated with the TLR signaling cascade (i.e., *Myd88*, RelA/p65 (regulatory subunit of NF-κB), *Tnf*α, *Il12b*, *Il6* and *Cox2*). *Tlr4* has been reported to regulate *Cox2* expression in DSS-induced colitis [59], which is similar to our results in Hi Tannin Black DSS rats.

Although we observe higher TLR signaling cascade expression in DSS rats, we do not observe extensive inflammatory cell infiltration or fold changes in relative expression of *Cox2*, *Il12b* and *IL6*, which are commonly associated with an overactive TLR/NF-κB signaling cascade in experimental colitis and UC patients. Furthermore, we observe no differences in activated NF-κB between healthy and diseased animals for any diet, and DSS-treated rats demonstrated no significant differences in inflammatory infiltration. Surprisingly, cellulose control rats have a significantly higher inflammation score than cellulose DSS rats.

To our knowledge, this is the first study to analyze the effects of sorghum bran-based diets on an experimental model of UC. We observe distinct differences among bran diets that contain 3-deoxyanthocyanins, condensed tannins or both compounds and a cellulose control diet. Since diets were prepared to contain similar levels of macronutrients and types of dietary fiber, results suggest that the presence of these dietary polyphenols may influence the luminal and colonic mucosal environments. Our results indicate that these sorghum bran diets can suppress symptoms, such as weight loss and bloody diarrhea, commonly reported in DSS colitis. We demonstrate that although these diets elevated basal injury scores, they may mitigate colonic injury and epithelial dysfunction induced during colitis by upregulating the proliferation and expression of *Tff3*, *Tgf*β and SCFA transporter expression. Further classification of the microbiome and a metabolomics profile will help elucidate the mechanisms by which bioactive compounds in sorghum bran may alter the luminal environment and mitigate UC.

## Figures and Tables

**Table 1:** Diet ingredients, sorghum bran proximate analysis, fiber characterization and the antioxidant capacity and phenol content of the mixed diets.

Ingredient/Composition	Cellulose	Black Bran	Sumac Bran	Hi Tannin Black Bran
INGREDIENT (g/kg) ^1^				
Dextrose	510.6	459.3	441.8	425.7
Casein	223.5	208.9	205.8	206.6
DL-methionine	3.4	3.4	3.4	3.4
Cellulose	60.0	0.00	0.00	0.00
Sorghum bran	0.00	130.3	162.4	174.9
Corn oil	150.0	145.8	134.6	137.1
Mineral mix, AIN-76A	39.1	39.0	38.8	38.9
Vitamin mix AIN-76A	11.2	11.2	11.1	11.2
Choline bitartrate	2.2	2.2	2.2	2.2
BRAN MACRONUTRIENT COMPOSITION (%) *				
Protein	-	12.17	11.24	10.18
Lipid	-	3.22	10.04	7.83
Fiber	-	52.98	41.58	38.31
Ash	-	3.37	4.94	2.81
Carbohydrate	-	14.87	20.44	30.04
Moisture	-	13.39	11.76	10.83
FIBER CHARACTERIZATION				
Soluble fiber, %	6.0	6.3	11.7	10.0
Insoluble fiber, %	94.0	94.0	88.3	90.0
ANTIOXIDANT CAPACITY AND PHENOLS				
ABTS ^†^	ND	64.2	182.4	136.3
Total phenols ^‡^	ND	5.4	11.6	8.8
Tannins ^‡^	ND	0.2	14.0	13.5

^1^ All ingredients acquired from Harlan (Houston, TX, USA), except the corn oil was acquired from DYETS. ND = not detected. * Measured in sorghum bran on an as-is basis. ^†^ Antioxidant capacity using the ABTS method (µmol Trolox equivalent (TE)/g diet). ^‡^ Total phenols (mg gallic acid equivalent (GAE)/g diet) and tannins (mg catechin equivalent (CE)/g diet).

**Table 2:** Sorghum brans elevated fecal moisture content, but DSS only increased fecal moisture content in rats consuming the Sumac bran diet after the first two DSS treatment periods.^1^

Time Relative to DSS	Sample Day	Cellulose		Black Bran		Sumac Bran		Hi Tannin Black Bran		*p*-Value		
		Control	DSS	Control	DSS	Control	DSS	Control	DSS	Diet	DSS	Diet*DSS
Pre DSS#1	38	39.81 ± 0.89 ^a^	38.87 ± 1.00 ^a^	47.60 ± 0.49 ^b^	46.47 ± 0.75 ^b^	52.25 ± 0.57 ^c^	53.05 ± 0.43 ^c^	48.40 ± 0.30 ^b^	47.89 ± 0.34 ^b^	<0.0001		
Post DSS#1	44	38.68 ± 0.83 ^a^	39.98 ± 0.75 ^a^	47.74 ± 0.20 ^b^	47.74 ± 0.20 ^b^	50.28 ± 0.55 ^c^	54.98 ± 1.43 ^d^	49.43 ± 0.92 ^b,c^	48.90 ± 0.65 ^b,c^	<0.0001	0.0163	0.006
Post DSS#2	58	40.41 ± 0.89 ^a^	41.53 ± 0.42 ^a^	47.91 ± 0.21 ^b^	48.28 ± 0.62 ^b^	51.41 ± 1.03 ^c^	53.79 ± 0.76 ^d^	49.63 ± 0.56 ^b,c^	50.34 ± 0.77 ^c^	<0.0001	0.0241	
Post DSS#3	81	43.25 ± 1.11 ^a^	40.89 ± 0.47 ^a^	48.05 ± 0.51 ^b^	49.57 ± 0.41 ^b,c^	53.23 ± 0.54 ^d^	55.61 ± 0.89 ^d^	50.06 ± 0.39 ^c^	49.97 ± 0.51 ^c^	<0.0001		0.003

^1^ Values are least square means ± standard error of the mean. DSS = dextran sodium sulfate, Diet*DSS = Diet and DSS interaction. ^a,b,c^ Means in a row without a common superscript differ (*p* < 0.05).

**Table 3:** DSS treatment significantly affected the proliferative index and crypt height.^1^

Variable	Cellulose		Black Bran		Sumac Bran		Hi Tannin Black Bran		*p*-Value		
	Control	DSS	Control	DSS	Control	DSS	Control	DSS	Diet	DSS	Diet*DSS
Proliferative index	9.59 ± 2.88 ^a,b^	9.35 ± 3.44 ^a,b^	8.15 ± 3.20 ^a^	11.18 ± 3.00 ^b^	9.83 ± 3.15 ^a,b^	10.75 ± 3.79 ^b^	9.17 ± 3.02 ^a,b^	10.89 ± 3.35 ^b^		0.0112	
Proliferative zone	36.96 ± 6.54 ^b^	34.45 ± 6.50 ^a,b^	31.49 ± 5.39 ^a^	35.18 ± 5.08 ^a,b^	36.29 ± 7.26 ^a,b^	37.52 ± 5.57 ^b^	34.91 ± 5.95 ^a,b^	37.72 ± 7.04 ^b^			
Apoptotic index	0.18 ± 0.01 ^a,b^	0.18 ± 0.01 ^a,b^	0.15 ± 0.01 ^a,b^	0.18 ± 0.01 ^a,b^	0.29 ± 0.01 ^b^	0.22 ± 0.01 ^a,b^	0.23 ± 0.01 ^a,b^	0.09 ± 0.01 ^a^			
Crypt height	30.00 ± 0.74 ^b^	27.73 ± 0.76 ^a^	30.74 ± 0.77 ^b^	26.62 ± 0.88 ^a^	32.02 ± 0.72 ^b^	27.08 ± 1.01 ^a^	31.23 ± 0.68 ^b^	26.29 ± 0.98 ^a^		<0.0001	

^1^ Values are least square means ± standard error of the mean. DSS = dextran sodium sulfate, Diet*DSS = Diet and DSS interaction. ^a,b,c^ Means in a row without a common superscript differ (*p* < 0.05).

**Table 4:** Twenty four hour SCFA excretion measured 38 and 81 days post rats arrival (µmol/24 h) in feces from rats treated with water (control) or DSS to induce colitis and consuming diets containing either cellulose or brans from Black, Sumac or Hi Tannin Black bran.^1^

SCFA		Cellulose		Black Bran		Sumac Bran		Hi Tannin Black Bran		*p*-Value		
		Control	DSS	Control	DSS	Control	DSS	Control	DSS	Diet	DSS	Diet*DSS
Day 38	Acetic	43.01 ± 6.70 ^a^	32.55 ± 6.34 ^a,b,c^	29.63 ± 2.76 ^b,c^	26.95 ± 2.16 ^c^	32.52 ± 2.97 ^a,b,c^	39.96 ± 5.52 ^a,b^	28.52 ± 2.05 ^c^	25.94 ± 2.59 ^c^	0.0299		
	Propionic	8.72 ± 0.92 ^c^	7.85 ± 1.11 ^c^	11.78 ± 1.22 ^b^	11.27 ± 1.20 ^b,c^	18.53 ± 1.42 ^a^	17.60 ± 1.82 ^a^	13.64 ± 0.86 ^b^	12.41 ± 1.01 ^b^	0.0001		
	Isobutyric	1.44 ± 0.16 ^b^	1.35 ± 0.13 ^b^	1.49 ± 0.13 ^b^	1.48 ± 0.15 ^b^	2.91 ± 0.30 ^a^	2.82 ± 0.28 ^a^	3.16 ± 0.18 ^a^	3.02 ± 0.27 ^a^	0.0001		
	Butyric	13.34 ± 2.54 ^a,b^	12.79 ± 2.30 ^a,b^	16.13 ± 1.17 ^a^	14.36 ± 1.08 ^a,b^	8.15 ± 0.77 ^c^	8.18 ± 0.63 ^c^	12.99 ± 0.85 ^ab^	11.84 ± 1.16 ^b,c^	0.0001		
	Isovaleric	2.89 ± 0.39 ^b^	2.74 ± 0.28 ^b^	3.70 ± 0.31 ^b^	3.65 ± 0.36 ^b^	7.73 ± 0.81 ^a^	7.49 ± 0.81 ^a^	8.46 ± 0.50 ^a^	8.18 ± 0.78 ^a^	0.0001		
	Valeric	4.40 ± 0.40 ^b^	4.17 ± 0.48 ^b^	5.60 ± 0.70 ^b^	5.55 ± 1.04 ^b^	1.75 ± 0.96 ^b^	1.50 ± 0.73 ^b^	8.39 ± 0.99 ^b^	16.52 ± 7.90 ^a^	0.0035		
	Total	73.80 ± 9.62 ^a^	61.46 ± 9.35 ^a^	68.33 ± 5.81 ^a^	63.26 ± 5.52 ^a^	71.59 ± 4.67 ^a^	81.32 ± 7.49 ^a^	75.16 ± 3.67 ^a^	77.90 ± 8.97 ^a^			
Day 81	Acetic	49.19 ± 6.70 ^a^	48.45 ± 6.33 ^a^	26.04 ± 2.76 ^b^	33.71 ± 2.17 ^a,b^	40.33 ± 2.98 ^a,b^	50.24 ± 5.52 ^a^	51.30 ± 2.05 ^b^	37.20 ± 2.59 ^a,b^			
	Propionic	10.79 ± 0.92 ^b^	12.59 ± 1.11 ^a,b^	9.10 ± 1.22 ^b^	22.43 ± 1.20 ^a^	22.40 ± 1.42 ^a^	23.98 ± 1.82 ^a^	22.06 ± 0.86 ^a^	17.14 ± 1.01 ^a,b^	0.0550		
	Isobutyric	1.52 ± 0.16 ^c^	1.53 ± 0.13 ^c^	1.14 ± 0.13 ^c^	1.54 ± 0.15 ^c^	3.75 ± 0.30 ^b^	3.77 ± 0.28 ^b^	5.85 ± 0.18 ^a^	4.53 ± 0.27 ^a,b^	0.0001		
	Butyric	19.67 ± 2.54 ^a^	16.45 ± 2.30 ^a,b^	15.98 ± 1.17 ^a,b^	20.07 ± 1.08 ^a^	9.20 ± 0.77 ^b^	8.80 ± 0.63 ^b^	20.24 ± 0.85 ^a^	14.08 ± 1.16 ^a,b^	0.0065		
	Isovaleric	2.83 ± 0.39 ^c^	2.94 ± 0.28 ^c^	2.99 ± 0.31 ^c^	3.78 ± 0.36 ^c^	9.48 ± 0.81 ^b^	9.28 ± 0.58 ^b^	14.91 ± 0.50 ^a^	11.35 ± 0.78 ^a,b^	0.0001		
	Valeric	4.17 ± 0.40 ^b^	4.46 ± 0.48 ^b^	3.98 ± 0.70 ^b^	5.04 ± 1.04 ^b^	0.71 ± 0.96 ^b^	0.55 ± 0.73 ^b^	14.39 ± 0.99 ^a^	12.11 ± 7.90 ^a^	0.0001		
	Total	88.08 ± 9.62 ^a,b^	86.41 ± 9.35 ^a,b^	59.22 ± 5.81 ^a^	86.55 ± 5.53 ^a,b^	85.88 ± 4.67 ^a,b^	96.61 ± 7.48 ^a,b^	128.74 ± 3.67 ^a^	96.41 ± 8.97 ^a,b^			

^1^ Values are least square means ± standard error of the mean. DSS = dextran sodium sulfate, Diet*DSS = Diet and DSS interaction. ^a,b,c^ Means in a row without a common superscript differ (*p* < 0.05).

## Supplementary Materials

The following are available online at www.mdpi.com/2072-6643/9/4/330/s1: Table S1: Assay ID for selected gene targets, Table S2: Mean body weight (g) of rats measured throughout the study, Table S3: Intake (g/24 h) of rats measured prior to DSS#1 (Day 34), following DSS#2 (Day 56) and following DSS#3 (Day 77), Table S4: Fecal SCFA excretion (µmol/24 h) measured on Days 44, 66 and 72 in rats treated with water (control) or DSS to induce colitis and consuming diets containing either cellulose or brans from Black, Sumac or Hi Tannin Black bran, Table S5: Relative expression of selected gene targets (2^−Δ*C*t^) in scraped colon mucosa from rats treated with water (control) or DSS to induce colitis and consuming diets containing either cellulose or brans from Black, Sumac or Hi Tannin Black sorghums as the fiber source.^1^ Expression levels were normalized to 18S gene expression.

## Abbreviations

DSS	Dextran sodium sulfate
IBD	Inflammatory bowel disease
NF-κB	Nuclear factor- κB
SCFA	Short chain fatty acids
TLR	Toll-like receptor
UC	Ulcerative colitis
