# Supplementary material for: Impact of Novel Sorghum Bran Diets on DSS-Induced Colitis
Source: Nutrients. 2017 Mar 27;9(4):330. doi: 10.3390/nu9040330 (PMC5409669; doi:10.3390/nu9040330)
Supplement: Supplementary file 1 [file nutrients-09-00330-s001.docx]

Supplementary Materials: Impact of Novel Sorghum Bran Diets on
DSS-Induced Colitis

Lauren E. Ritchie, Stella S. Taddeo, Brad R. Weeks, Raymond J. Carroll, Linda Dykes, Lloyd W. Rooney and Nancy D. Turner

**Table S1.** Assay ID for selected gene targets.

| **Gene Symbol** | **Alias** | **Assay ID** |
| --- | --- | --- |
| *Tollip* |  | Rn01479669_m1 |
| *Tff3* | ITF, TREFOIL | Rn00564851_m1 |
| *Tjp1* | *Zo1* | Rn02116071_s1 |
| *Slc16a1* | MCT1 | Rn00562332_m1 |
| *Il6* |  | Rn00566707_m1 |
| *Il1b* |  | Rn01514151_m1 |
| *Il12b* |  | Rn00575112_m1 |
| *Tnf* | Tnfα | Rn00562055_m1 |
| *Slc5a8* |  | Rn01503812_m1 |
| *Tgfb* | Tgfβ | Rn01442102_m1 |
| *Tlr4* |  | Rn00569848_m1 |
| *Tlr2* |  | Rn02133647_s1 |
| *Myd88* |  | Rn01640052_g1 |
| *Rel/p65* | NFκB | Rn01502266_m1 |
| *Ptgs2* | *Cox2* | Rn01483828_m1 |

**Table S2**. Mean body weight (g) of rats measured throughout the study.^1^

| **Time point** | **Date** | **Cellulose** | | **Black Bran** | | **Sumac Bran** | | **Hi Tannin Black Bran** | | ***P*-value** | | |
| --- | --- | --- | --- | --- | --- | --- | --- | --- | --- | --- | --- | --- |
|  |  | **Control** | **DSS** | **Control** | **DSS** | **Control** | **DSS** | **Control** | **DSS** | **Diet** | **DSS** | **Diet*DSS** |
| Diet start | Day 19 | 156.1 ± 1.89^a^ | 155.4 ± 1.97^a^ | 157.5 ± 1.89^a^ | 160.1 ± 1.91^a^ | 159.3 ± 1.65^a^ | 160.1 ± 1.65^a^ | 161.8 ± 1.50^a^ | 157.3 ± 1.48^a^ |  |  |  |
| Intake #1 | Day 37 | 255.7 ± 5.32^a^ | 261.5 ± 3.58^ab^ | 261.5 ± 3.54^ab^ | 264.8 ± 2.84^ab^ | 267.5 ± 5.03^ab^ | 262.6 ± 4.23^ab^ | 268.7 ± 4.05^b^ | 260.2 ± 4.46^ab^ |  |  |  |
| Pre-DSS #1 | Day 39 | 269.5 ± 5.80^a^ | 275.0 ± 3.34^ab^ | 273.8 ± 3.71^ab^ | 279.4 ± 2.65^ab^ | 279.4 ± 4.40^ab^ | 277.2 ± 4.94^ab^ | 283.2 ± 4.45^b^ | 273.8 ± 4.40^ab^ |  |  |  |
| Pre-DSS #2 | Day 53 | 318.1 ± 6.66^a^ | 324.8 ± 5.25^ab^ | 321.7 ± 5.94^ab^ | 329.0 ± 4.04^ab^ | 333.2 ± 7.06^ab^ | 322.3 ± 6.38^ab^ | 337.9 ± 5.74^b^ | 324.0 ± 7.16^ab^ |  |  |  |
| Intake #2 | Day 58 | 329.4 ± 6.59^a^ | 334.7 ± 5.65^ab^ | 332.6 ± 6.73^ab^ | 340.3 ± 4.52^ab^ | 344.9 ± 7.55^ab^ | 332.5 ± 6.31^ab^ | 348.6 ± 5.93^b^ | 335.3 ± 8.26^ab^ |  |  |  |
| Pre-DSS #3 | Day 67 | 349.7 ± 7.60^a^ | 356.3 ± 7.04^a^ | 353.1 ± 7.53^a^ | 361.6 ± 4.74^a^ | 365.2 ± 7.95^a^ | 353.8 ± 7.01^a^ | 367.9 ± 6.28^a^ | 356.5 ± 9.04^a^ |  |  |  |
| Intake #3 | Day 77 | 369.6 ± 9.05^a^ | 376.5 ± 7.85^a^ | 373.7 ± 8.04^a^ | 382.3 ± 5.63^a^ | 386.4 ± 8.89^a^ | 372.3 ± 8.28^a^ | 390.3 ± 8.11^a^ | 376.6 ± 9.92^a^ |  |  |  |
| Termination | Day 82 | 374.3 ± 8.86^a^ | 382.2 ± 7.81^a^ | 379.0 ± 7.95^a^ | 387.0 ± 5.41^a^ | 391.2 ± 8.90^a^ | 374.9 ± 8.43^a^ | 394.8 ± 8.08^a^ | 381.5 ± 9.94^a^ |  |  |  |

^1^ Values are LS means ± SEM. DSS = dextran sodium sulfate. Diet*DSS = Diet and DSS interaction. ^a,b,c^ Means in a row without a common superscript differ (*p* < 0.05).

**Table S3.** Intake (g/24 h) of rats measured prior to DSS#1 (Day 34), following DSS#2 (Day 56) and following DSS#3 (Day 77).^1^

| **Time point** | **Date** | **Cellulose** | | **Black Bran** | | **Sumac Bran** | | **Hi Tannin Black Bran** | | ***P*-value** | | |
| --- | --- | --- | --- | --- | --- | --- | --- | --- | --- | --- | --- | --- |
|  |  | **Control** | **DSS** | **Control** | **DSS** | **Control** | **DSS** | **Control** | **DSS** | **Diet** | **DSS** | **Diet*DSS** |
| Pre DSS #1 | Day 33 | 19.0 ± 0.7^ab^ | 18.1 ± 0.7^a^ | 19.6 ± 0.7^ab^ | 19.1 ± 0.7^ab^ | 19.6 ± 0.7^ab^ | 20.0 ± 0.7^b^ | 19.5 ± 0.7^ab^ | 18.9 ± 0.7^ab^ |  |  |  |
| Post DSS #2 | Day 56 | 19.6 ± 0.6^a^ | 19.1 ± 0.6^a^ | 19.5 ± 0.6^a^ | 19.9 ± 0.6^a^ | 20.2 ± 0.6^a^ | 19.5 ± 0.6^a^ | 19.3 ± 0.6^a^ | 19.2 ± 0.6^a^ |  |  |  |
| Post DSS #3 | Day 77 | 21.5 ± 0.7^a^ | 21.3 ± 0.7^a^ | 20.5 ± 0.7^a^ | 20.4 ± 0.7^a^ | 21.0 ± 0.7^a^ | 19.9 ± 0.7^a^ | 21.2 ± 0.7^a^ | 20.0 ± 0.7^a^ |  |  |  |

^1^ Values are LS means ± SEM. DSS = dextran sodium sulfate. Diet * DSS = Diet and DSS interaction. ^a,b,c^ Means in a row without a common superscript differ (*p* < 0.05).

**Table S4.** Fecal SCFA excretion (µmol/24 h) measured on Days 44, 66 and 72 in rats treated with water (control) or DSS to induce colitis and consuming diets containing either cellulose or brans from Black, Sumac or Hi Tannin Black bran.^1^

|  | | **Cellulose** | | **Black bran** | | **Sumac bran** | | **Hi Tannin Black bran** | | ***P*-value** | | |
| --- | --- | --- | --- | --- | --- | --- | --- | --- | --- | --- | --- | --- |
|  |  | **Control** | **DSS** | **Control** | **DSS** | **Control** | **DSS** | **Control** | **DSS** | **Diet** | **DSS** | **Diet*DSS** |
| Day 44 | Acetic | 89.24 ± 6.70^b^ | 60.11 ± 6.34^ab^ | 51.17 ± 2.76^a^ | 61.65 ± 2.17^ab^ | 67.89 ± 2.98^ab^ | 68.91 ± 5.52^ab^ | 46.41 ± 2.05^a^ | 53.54 ± 2.59^a^ |  |  |  |
|  | Propionic | 18.57 ± 0.92^a^ | 23.40 ± 1.11^ab^ | 20.51 ± 1.22^a^ | 27.98 ± 1.20^abc^ | 37.32 ± 1.42^c^ | 33.40 ± 1.82^bc^ | 22.03 ± 0.86^a^ | 32.80 ± 1.00^bc^ | 0.0040 |  |  |
|  | Isobutyric | 2.45 ± 0.16^a^ | 3.55 ± 0.13^ab^ | 2.34 ± 0.13^a^ | 2.91 ± 0.15^a^ | 6.58 ± 0.30^c^ | 4.75 ± 0.28^b^ | 4.96 ± 0.18^bc^ | 6.46 ± 0.27^c^ | <0.0001 |  | 0.0300 |
|  | Butyric | 34.47 ± 2.54^c^ | 22.26 ± 2.30^abc^ | 28.18 ± 1.17^bc^ | 22.74 ± 1.08^abc^ | 16.99 ± 0.77^ab^ | 13.02 ± 0.63^a^ | 22.70 ± 0.85^abc^ | 22.78 ± 1.16^abc^ | 0.0235 |  |  |
|  | Isovaleric | 4.67 ± 0.39^a^ | 7.58 ± 0.28^a^ | 5.86 ± 0.31^a^ | 7.62 ± 0.36^a^ | 17.21 ± 0.81^c^ | 12.69 ± 0.58^b^ | 12.81 ± 0.50^b^ | 15.91 ± 0.78^bc^ | <0.0001 |  |  |
|  | Valeric | 7.60 ± 0.40^b^ | 9.23 ± 0.48^b^ | 7.45 ± 0.70^b^ | 8.00 ± 1.04^b^ | 1.38 ± 0.96^a^ | 0.96 ± 0.73^a^ | 12.99 ± 0.99^c^ | 17.54 ± 7.90^d^ | <0.0001 |  |  |
|  | Total | 157.0 ± 9.62^a^ | 126.13 ± 9.35^a^ | 115.52 ± 5.81^a^ | 130.92 ± 5.53^a^ | 147.36 ± 4.67^a^ | 133.73 ± 7.49^a^ | 121.89 ± 3.70^a^ | 149.03 ± 8.97^a^ |  |  |  |
| Day 66 | Acetic | 48.15 ± 6.70^b^ | 29.06 ± 6.34^a^ | 26.08 ± 2.76^a^ | 35.66 ± 2.17^ab^ | 33.01 ± 2.98^a^ | 39.14 ± 5.52^ab^ | 30.68 ± 2.05^a^ | 31.63 ± 2.59^a^ |  |  | 0.0300 |
|  | Propionic | 10.73 ± 0.92^ab^ | 6.61 ± 1.11^a^ | 9.81 ± 1.22^ab^ | 12.98 ± 1.20^b^ | 19.39 ± 1.42^c^ | 19.36 ± 1.82^c^ | 14.09 ± 0.86^b^ | 13.57 ± 1.01^ab^ | <0.0001 |  |  |
|  | Isobutyric | 1.54 ± 0.16^a^ | 1.28 ± 0.13^a^ | 1.23 ± 0.13^a^ | 1.53 ± 0.15^a^ | 3.53 ± 0.30^b^ | 3.13 ± 0.28^b^ | 3.47 ± 0.18^b^ | 3.80 ± 0.27^b^ | <0.0002 |  |  |
|  | Butyric | 17.24 ± 2.54^bc^ | 10.29 ± 2.30^a^ | 16.61 ± 1.17^bc^ | 20.55 ± 1.08^c^ | 8.43 ± 0.77^a^ | 7.09 ± 0.63^a^ | 12.54 ± 0.85^ab^ | 10.07 ± 1.16^a^ | <0.0003 |  |  |
|  | Isovaleric | 2.75 ± 0.39^a^ | 2.69 ± 0.28^a^ | 2.92 ± 0.31^a^ | 3.63 ± 0.36^a^ | 8.95 ± 0.81^b^ | 7.72 ± 0.58^b^ | 8.67 ± 0.50^b^ | 9.50 ± 0.78^b^ | <0.0004 |  |  |
|  | Valeric | 4.16 ± 0.40^abc^ | 11.01 ± 0.48^bc^ | 4.14 ± 0.70^abc^ | 4.90 ± 1.04^abc^ | 0.53 ± 0.96^a^ | 0.65 ± 0.73^a^ | 9.04 ± 0.99^b^ | 10.14 ± 7.90^b^ | 0.0127 |  |  |
|  | Total | 84.57 ± 9.62^a^ | 60.94 ± 9.35^a^ | 60.79 ± 5.81^a^ | 79.23 ± 5.53^a^ | 73.84 ± 4.67^a^ | 77.09 ± 7.50^a^ | 78.49 ± 3.67^a^ | 78.69 ± 8.97^a^ |  |  |  |
| Day 72 | Acetic | 31.82 ± 6.70^bc^ | 24.94 ± 6.34^c^ | 21.92 ± 2.76^ab^ | 22.07 ± 2.17^bc^ | 26.70 ± 2.98^a^ | 31.05 ± 5.52^c^ | 28.89 ± 2.05^c^ | 28.80 ± 2.59^c^ |  |  |  |
|  | Propionic | 7.99 ± 0.92^a^ | 9.58 ± 1.11^ab^ | 9.77 ± 1.22^ab^ | 12.65 ± 1.20^abc^ | 17.64 ± 1.42^c^ | 18.04 ± 1.82^c^ | 13.69 ± 0.86^abc^ | 15.29 ± 1.01^bc^ | 0.0006 |  |  |
|  | Isobutyric | 1.22 ± 0.16^a^ | 1.38 ± 0.13^a^ | 1.16 ± 0.13^a^ | 1.38 ± 0.15^a^ | 3.06 ± 0.30^bc^ | 2.67 ± 0.28^b^ | 3.56 ± 0.18^bc^ | 3.73 ± 0.27^c^ | <0.0001 |  |  |
|  | Butyric | 15.96 ± 2.54^c^ | 8.53 ± 2.30^ab^ | 15.31 ± 1.17^c^ | 14.75 ± 1.08^c^ | 7.50 ± 0.77^ab^ | 5.85 ± 0.63^a^ | 12.34 ± 0.85^bc^ | 11.56 ± 1.16^abc^ | 0.0020 |  |  |
|  | Isovaleric | 2.03 ± 0.39^a^ | 2.67 ± 0.28^a^ | 2.60 ± 0.31^a^ | 3.09 ± 0.37^a^ | 7.50 ± 0.81^bc^ | 6.13 ± 0.58^b^ | 8.73 ± 0.50^c^ | 8.84 ± 0.78^c^ | <0.0001 |  |  |
|  | Valeric | 3.69 ± 0.40^b^ | 3.56 ± 0.48^b^ | 3.85 ± 0.70^b^ | 4.31 ± 1.04^b^ | 0.60 ± 0.96^a^ | 0.32 ± 0.73^a^ | 9.41 ± 0.99^c^ | 9.56 ± 7.90^c^ | <0.0001 |  |  |
|  | Total | 62.71 ± 9.62^a^ | 50.67 ± 9.35^a^ | 54.61 ± 5.81^a^ | 63.25 ± 5.53^a^ | 63.00 ± 4.67^a^ | 64.07 ± 7.50^a^ | 76.61 ± 3.67^a^ | 77.78 ± 8.97^a^ |  |  |  |

^1^ Values are LS means ± SEM. DSS = dextran sodium sulfate. Diet * DSS = Diet and DSS interaction. ^a,b,c^ Means in a row without a common superscript differ (*p* < 0.05).

**Table S5.** Relative expression of selected gene targets (2^−Δ^*^C^*^t^) in scraped colon mucosa from rats treated with water (control) or DSS to induce colitis and consuming diets containing either cellulose or brans from Black, Sumac or Hi Tannin Black sorghums as the fiber source.^1^ Expression levels were normalized to 18S gene expression.

|  | **Cellulose** | | **Black Bran** | | **Sumac Bran** | | **Hi Tannin Black Bran** | | ***P*-value** | | |
| --- | --- | --- | --- | --- | --- | --- | --- | --- | --- | --- | --- |
|  | **Control** | **DSS** | **Control** | **DSS** | **Control** | **DSS** | **Control** | **DSS** | **Diet** | **DSS** | **Diet*DSS** |
| *Tlr2* | 2.21 ± 0.5^ab^ | 2.11 ± 0.57^ab^ | 0.83 ± 0.11^a^ | 3.29 ± 1.75^ab^ | 1.94 ± 0.93^ab^ | 4.25 ± 2.01^ab^ | 1.06 ± 0.29^ab^ | 5.46 ± 2.32^b^ |  | 0.0180 |  |
| *Tlr4* | 23.72 ± 4.59^ab^ | 19.98 ± 3.67^a^ | 18.22 ± 2.73^a^ | 50.10 ± 23.04^ab^ | 36.45 ± 10.06^ab^ | 41.92 ± 11.75^ab^ | 20.10 ± 3.77^a^ | 56.34 ± 18.18^b^ |  | 0.0440 |  |
| *Myd88* | 36.81 ± 8.93^ab^ | 24.77 ± 3.93^ab^ | 23.64 ± 3.01^a^ | 68.64 ± 28.63^bc^ | 30.46 ± 5.17^ab^ | 47.81 ± 11.56^abc^ | 23.65 ± 4.77^ab^ | 82.95 ± 24.48^c^ |  | 0.0100 |  |
| *RelA/p65* | 47.13 ± 11.33^ab^ | 38.61 ± 5.62^ab^ | 35.06 ± 4.13^ab^ | 81.66 ± 25.60^bc^ | 45.45 ± 6.65^ab^ | 80.52 ± 20.81^bc^ | 33.47 ± 6.44^ab^ | 112.18 ± 34.75^c^ |  | 0.0039 |  |
| *Tollip* | 57.24 ± 12.44^ab^ | 50.18 ± 9.85^ab^ | 31.38 ± 5.03^ab^ | 99.09 ± 32.07^bc^ | 51.2 ± 11.00^ab^ | 117.27 ± 41.50^bc^ | 35.22 ± 7.38^ab^ | 158.14 ± 62.81^c^ |  | 0.0039 |  |
| *Tnfα* | 1.68 ± 0.42^ab^ | 1.84 ± 0.43^ab^ | 1.54 ± 0.25^a^ | 3.32 ± 0.78^abc^ | 1.60 ± 0.42^ab^ | 4.30 ± 1.62^c^ | 1.18 ± 0.28^a^ | 3.80 ± 1.03^bc^ |  | 0.0020 |  |
| *Cox-2* | 6.41 ± 1.33^a^ | 5.01 ± 0.87^a^ | 4.12 ± 0.72^a^ | 8.81 ± 2.87^a^ | 5.80 ± 1.43^a^ | 8.18 ± 2.28^a^ | 4.92 ± 0.86^a^ | 17.36 ± 4.72^b^ |  | 0.0060 | 0.0260 |
| *Tff3* | 647.6 ± 149.6^ab^ | 540.8 ± 89.2^a^ | 442.6 ± 51.9^a^ | 1226.5 ± 515.3^ab^ | 629.7 ± 159.8^ab^ | 992.0 ± 302.8^ab^ | 410.3 ± 89.4^a^ | 1492.9 ± 548.7^b^ |  | 0.0152 |  |
| *Tgfβ* | 10.95 ± 3.03^a^ | 7.61 ± 1.20^a^ | 8.22 ± 1.16^a^ | 16.42 ± 5.93^a^ | 6.86 ± 1.17^a^ | 18.09 ± 6.23^a^ | 8.7 ± 2.24^a^ | 33.53 ± 11.12^b^ |  | 0.0070 |  |
| *Il-12b* | 0.12 ± 0.03^a^ | 0.14 ± 0.03^a^ | 0.13 ± 0.03^a^ | 0.20 ± 0.05^a^ | 0.15 ± 0.03^a^ | 0.27 ± 0.07^a^ | 0.08 ± 0.02^a^ | 0.50 ± 0.15^b^ |  | 0.0017 | 0.0170 |
| *Il-1b* | 83.64 ± 67.48^b^ | 0.74 ± 0.23^a^ | 0.69 ± 0.20^a^ | 2.89 ± 2.23^a^ | 0.74 ± 0.32^a^ | 1.22 ± 0.30^a^ | 0.59 ± 0.17^a^ | 3.62 ± 1.36^a^ |  |  |  |
| *Il-6* | 0.89 ± 0.21^a^ | 0.66 ± 0.17^a^ | 0.56 ± 0.11^a^ | 1.29 ± 0.53^ab^ | 0.72 ± 0.21^a^ | 2.10 ± 0.83^b^ | 0.48 ± 0.13^a^ | 2.09 ± 0.61^b^ |  | 0.0042 |  |
| *Slc16a* | 146.45 ± 30.3^a^ | 96.03 ± 16.71^a^ | 98.19 ± 13.53^a^ | 201.18 ± 54.23^ab^ | 104.71 ± 22.81^a^ | 219.38 ± 68.19^ab^ | 88.22 ± 19.07^a^ | 311.93 ± 111.45^b^ |  | 0.0102 |  |
| *Slc5a8* | 56.27 ± 13.37^a^ | 50.47 ± 10.53^a^ | 35.53 ± 5.43^a^ | 105.49 ± 50.06^ab^ | 62.34 ± 18.78^a^ | 119.80 ± 44.07^ab^ | 35.83 ± 7.45^a^ | 156.88 ± 68.24^b^ |  | 0.0162 |  |

Values are LS means ± SEM. DSS = dextran sodium sulfate. Diet*DSS = Diet and DSS interaction. ^a,b,c^ Means in a row without a common superscript differ (*p* < 0.05).
